# Laser ablation of silicon with THz bursts of femtosecond pulses

**DOI:** 10.1038/s41598-021-92645-7

**Published:** 2021-06-25

**Authors:** Caterina Gaudiuso, Pavel N. Terekhin, Annalisa Volpe, Stefan Nolte, Bärbel Rethfeld, Antonio Ancona

**Affiliations:** 1 Department of Physics, University of Bari “Aldo Moro”, 70126 Bari, Italy; 2 CNR-IFN UOS BARI, Via Amendola 173, Bari, Italy; 3 Department of Physics and Research Center OPTIMAS, Technische Universität Kaiserslautern, Erwin-Schrödinger-Strasse 46, 67663 Kaiserslautern, Germany; 4 Institute of Applied Physics, Abbe Center of Photonics, Friedrich-Schiller-Universität Jena, Albert-Einstein-Strasse 15, 07745 Jena, Germany; 5 Fraunhofer Institute for Applied Optics and Precision Engineering IOF, Center of Excellence in Photonics, Albert-Einstein-Strasse 7, 07745 Jena, Germany

**Keywords:** Ultrafast lasers, Laser material processing

## Abstract

In this work, we performed an experimental investigation supported by a theoretical analysis, to improve knowledge on the laser ablation of silicon with THz bursts of femtosecond laser pulses. Laser ablated craters have been created using 200 fs pulses at a wavelength of 1030 nm on silicon samples systematically varying the burst features and comparing to the normal pulse mode (NPM). Using bursts in general allowed reducing the thermal load to the material, however, at the expense of the ablation rate. The higher the number of pulses in the bursts and the lower the intra-burst frequency, the lower is the specific ablation rate. However, bursts at 2 THz led to a higher specific ablation rate compared to NPM, in a narrow window of parameters. Theoretical investigations based on the numerical solution of the density-dependent two temperature model revealed that lower lattice temperatures are reached with more pulses and lower intra-burst frequencies, thus supporting the experimental evidence of the lower thermal load in burst mode (BM). This is ascribed to the weaker transient drop of reflectivity, which suggests that with bursts less energy is transferred from the laser to the material. This also explains the trends of the specific ablation rates. Moreover, we found that two-photon absorption plays a fundamental role during BM processing in the THz frequency range.

## Introduction

Ultrafast laser micromachining is typically characterized by superior quality and control^1–6^, however, at the expense of limited processing speed. In contrast, when fully exploiting the high repetition rates, pulse energies and average powers available today from many commercial femtosecond or picosecond laser sources, micromachining is often characterized by poor quality of the machined parts. This is attributed to the creation of heat cumulative effects that produce micro-cracks during the processing of brittle dielectric materials, like e.g. crystals or ceramics, or resolidified melt layers and large heat affected zones in the case of metals or semiconductors. Burst mode (BM) processing, i.e. using packets of laser pulses, has been proposed as a possible solution to reduce such thermal side effects. In the last years, several research studies have investigated ultrafast laser ablation using bursts with very different intra-burst pulse-to-pulse delays, from a few picoseconds^7,8^ to some microseconds^9–12^. BM processing has been found to be beneficial for many applications like e.g. surface structuring^13–16^, multi-photon absorption enhancement^17^, and laser milling^9,10,18^. Despite the recent advances, there are still open questions on the physical mechanisms that come into play and influence the laser–matter interaction, when bursts of ultrashort pulses are used instead of single pulses, the latter known as normal pulse mode (NPM) irradiation.

Silicon has been one of the first and most investigated materials for BM processing^19–22^. Recently, it has been shown that when laser milling silicon using bursts of 28 pulses at the intra-burst frequency of 154 MHz, a 2.1 times higher ablation efficiency can be achieved compared to the NPM case^11^. The authors ascribed this result to the establishment of a thermal ablation regime, similarly to what happens when using nanosecond pulse durations. However, their hypothesis has not been supported by any theoretical model. The same intra-burst time delay range was experimentally investigated by Neuenschwander et al.^23^. They also reported on a general enhancement of the maximum specific removal rate in BM processing, especially with a number of pulses in the burst up to 8 at the intra-burst frequency of 84 MHz. Moreover, it was found that reducing the number of pulses in the bursts to 2 and the intra-burst frequency down to 12 MHz the maximum specific removal rate decreases, as well. The authors supported this result with calorimetric measurements that showed that the samples micro-milled with a higher number of pulses in the bursts have better absorbance to laser radiation. However, a physical model explaining how the optical properties of a material may change upon irradiation with bursts of laser pulses is still lacking. Metzner et al.^24^ investigated the laser ablation of craters on silicon and cemented tungsten carbide substrates using bursts of a variable number of pulses and intra-burst repetition rate of 80 MHz. An enhancement of the ablated volume per pulse in the single burst, when the number of pulses was increased up to 5 was found. Nonetheless, a further raise of the number of pulses caused the ablated volume to decrease at an even number of pulses, differently from what was observed by Neuenschwander et al.^23^. Two effects were supposed to be responsible for such trend, namely plasma shielding and redeposition of ablated particles. Numerical simulations based on the solution of the classical heat conduction equation in case of a thermal point source, enabled to predict the ablation depth as a function of the number of pulses in the bursts. These results found a good agreement with the experimentally observed increase of the ablated volume up to 5 pulses, due to heat accumulation. However, such numerical model failed to describe the decrease of the ablated volume when an even number of pulses above 4 was exploited.

The beneficial effect of BM processing in terms of higher material removal rate has been also observed by Kerse et al.^25^. In particular, they investigated the role of the time delay between consecutive pulses, finding that a significant increase in the ablated volume of craters generated on silicon with bursts of 25 pulses was achieved, when the intra-burst repetition rate was increased from 1 MHz up to 108 MHz, at fixed pulse energy. However, a saturation of the material removal rate was observed by further increasing the intra-burst repetition rate probably due to plasma and particle shielding. The proposed solution to prevent such shielding effects and restore a high ablation rate was to decrease the pulse energy thereby increasing the number of pulses and the intra-burst repetition rate so that the total incident energy and duration of the burst would not change. An example is the use of a bust of 800 pulses at 3456 MHz instead of a burst of 25 pulses at 108 MHz with 32 times higher pulse energy. In that work, the role of the so-called ablation-cooling, namely the process of extracting thermal energy from the irradiated target by physically carrying away material through ablation, was highlighted. Typically, ablation-cooling occurs when the intra-burst delay *∆t* is shorter than the time needed for heat to diffuse out the irradiated area, so that the ablation-induced heat extraction is comparable or higher than the thermal diffusion. When such condition was met, the ablated volume was shown to increase with the number of pulses. In support of the experimental results, the authors developed a toy model that takes into account the repetitive heating and cooling cycles experienced by the material during the irradiation with bursts, which confirmed that the temperature reached inside the target was lower for higher repetition rates. Moreover, the model well fitted the experimental data of the ablated volume as a function of the total incident energy for all the investigated repetition rates up to the maximum one, i.e. 3456 MHz, but did not explicitly address the dependence of the ablated volume on the number of pulses.

All the previously mentioned studies dealt with bursts with repetition rates of a few GHz at most. So far very few works have investigated bursts in the THz frequency domain^8,21,22,26^, where the temporal separation between the pulses is of the order of the electron–phonon coupling time. The aim of this work is to fill this gap and study the laser ablation process of silicon using bursts of pulses with such an almost unexplored intra-burst frequency range. A recently developed method based on an array of birefringent crystals^7,8,21,22,27,28^ was used to generate ultrashort bursts of up to 16 pulses having an intra-burst repetition rate ranging from 250 GHz to 2 THz. Any potential reduction of the thermal load to the substrate and/or variations of the material removal rate was studied. The influence of the number of pulses within the bursts and their time delay on the specific ablation rate has been systematically investigated. The experimental results were supported by theoretical investigations of the evolution of the silicon sample properties during irradiation with a burst of ultrashort laser pulses through the numerical solution of the density-dependent two temperature model. Simulation results allowed understanding how the thermo-optical properties of silicon change during irradiation with bursts of pulses with THz frequencies providing useful indications to interpret the experimental data.

## Materials and methods

### Experimental set-up

An ultrafast laser source Pharos-SP from Light Conversion Ltd. delivering a linearly polarized beam at a wavelength of 1030 nm and a pulse duration of 200 fs has been used for the ablation experiments. The maximum available average power and pulse energy were 6 W and 1.5 mJ, respectively, while the repetition rate could be tuned between 1 kHz and 1 MHz. Moreover, a pulse picker allowed to select the number of pulses exiting the laser source.

A calcite birefringent crystals-based burst generator^27,28^ enabled to create bursts of up to 64 sub-pulses, with intra-burst delay time ranging from 0.5 to 4 ps, corresponding to intra-burst repetition rates from 2 THz down to 250 GHz. To this aim, the first crystal had a thickness of 0.9 mm. The thicknesses of the other four crystals were chosen to be multiples of 0.9 mm to be able to generate bursts of pulses with constant intra-burst frequency.

The laser beam from the burst generator had a diameter of around 4 mm. It was directed towards a focusing lens (focal length 100 mm), mounted on a computer controlled motorized axis (Aerotech, ANT130) which allowed the precise positioning of the beam focus on the targets, placed on a XY motorized translation stage (Aerotech ANT130). Crystalline p-doped silicon wafers with resistivity of 3–10 Ω cm and crystal orientation of [100] were used.

The ablation experiments were performed using a fixed number of 75 consecutive bursts at the laser repetition rate of 60 kHz. The total burst energy was varied between 2 and 40 µJ. Each burst was composed of a number *n* of 200-fs sub-pulses, which was varied between 2 and 16. The intra-burst frequency *f* was varied from 250 GHz up to 2 THz. For comparison, analogous experiments were carried out in normal pulse mode, i.e. not splitting the pristine pulses coming from the laser source, also referred to in the following as the *n* = 1 case. Table 1 summarizes the parameters used for ablation tests.

**Table 1:** Summary of experimental parameters.

Number of bursts, N	**75**			
Number of sub-pulses within the bursts, *n*	2	4	8	16
Intra-burst repetition rate, *f* (THz)	0.25–0.5–2	0.25–0.5–2	0.25–0.5–2	0.5–2
Burst energy, *E*_b_ (µJ)	2–3–4–5–6–8–10–16–20–32–40			

For each combination of parameters, five craters were created on the sample surfaces, in order to determine average values and variances of the crater diameters and depths. An optical microscope Nikon Eclipse ME600 and an optical ContourGT InMotion profilometer were exploited for measuring the craters’ diameters and depths, respectively.

### The density-dependent two temperature model applied to burst mode processing

Theoretical investigations of the evolution of the silicon sample irradiated by an ultrashort laser pulse in BM were performed within the framework of the density-dependent Two Temperature Model (nTTM) described in detail in Refs.^29–31^. This model was applied for the first time by van Driel^32^ and extended to account for transient optical parameters in Ref.^29^. The main difference between the well-known TTM^33,34^, usually used for the description of laser-metal interaction, and nTTM is that for laser-excited semiconductors the free carrier density has to be evaluated in an explicit way, because its value can be increased by orders of magnitude during irradiation. Therefore, the nTTM describes the evolution of free carrier density, carrier and lattice temperatures, particle and energy transport under the condition of transient optical properties.

In the framework of the nTTM the evolution of the carrier density is affected by the interplay of different processes during and after laser irradiation. It can be written as.

1 $$\frac{\partial {n}_{e}}{\partial t}=\frac{{\alpha }_{\text{SPA}}{I}_{\text{abs}}}{\hbar {\omega }_{L}}+\frac{{\beta }_{\text{TPA}}{I}_{\text{abs}}^{2}}{2\hbar {\omega }_{L}}+\delta {n}_{e}-\gamma {n}_{e}^{3}-\nabla \cdot \mathbf{J},$$

where on the right-hand side the first two terms are responsible for single and two photon absorption, the third term describes impact ionization, while the fourth one is an Auger recombination, and, finally, the last term stands for carrier transport with $\mathbf{J}$ being the electron–hole current density. The value ${I}_{\text{abs}}$ describes an absorbed laser pulse intensity at the photon energy $\hbar {\omega }_{L}$, whereas ${\alpha }_{\text{SPA}}$ and ${\beta }_{\text{TPA}}$ are the single and two photon absorption coefficients, respectively, $\delta$ and $\gamma$ are the coefficients for impact ionization and Auger recombination. We note that we neglect other possible recombination effects within the silicon sample. All the parameters used in the current work are taken from Ref.^29^, except optical properties at 1030 nm laser wavelength presented in Table 2.

**Table 2:** Model optical parameters for silicon at 1030 nm laser wavelength.

Quantity	Symbol	Value	References
Single photon absorption coefficient	${\alpha }_{\text{SPA}}$	$f\left(\hbar {\omega }_{L};{T}_{ph}\right)$	^35^
Two photon absorption coefficient	${\beta }_{\text{TPA}}$	2 cm/GW	^32^
Intrinsic dielectric constant	${\varepsilon }_{r}$	$12.709+0.0017146\cdot i$	^36^

Modification of the electron–hole energy density is ruled by carrier transport, carrier–phonon coupling and laser energy absorption:

2 $$\frac{\partial {U}_{e-h}}{\partial t}=-\nabla \cdot \mathbf{W}-G\left({T}_{e}-{T}_{ph}\right)+\left({\alpha }_{\text{SPA}}+{\alpha }_{\text{FCA}}\right){I}_{\text{abs}}+{\beta }_{\text{TPA}}{I}_{\text{abs}}^{2}.$$

$G$ is the carrier–phonon coupling factor often described as $G={C}_{e-h}/{\tau }_{\text{e}-\text{ph}}^{\text{relax}}$^29^, where the carrier heat capacity ${C}_{e-h}$ depends on the carrier temperature and density, whereas the carrier–phonon energy-relaxation time ${\tau }_{\text{e}-\text{ph}}^{\text{relax}}$ is taken as 0.5 ps^29,37,38^. ${T}_{e}$ is the electron temperature and ${T}_{ph}$ is the phonon temperature. A more detailed description of the heat current density $\mathbf{W}$ and the electron–hole current density $\mathbf{J}$ is given in Ref.^29^. The third and fourth terms in Eq. (2) describe laser energy absorption, where the coefficient ${\alpha }_{\text{FCA}}$ stands for the free carrier absorption.

The evolution of the lattice energy density can be represented as

3 $$\frac{\partial {U}_{ph}}{\partial t}=\nabla \cdot \left({\kappa }_{ph}\nabla {T}_{ph}\right)+G\left({T}_{e}-{T}_{ph}\right),$$

where the first term describes the energy transport with $\kappa _{{ph}}$ being the lattice thermal conductivity^29^ and the second one stands for the carrier–phonon energy exchange. The lattice and electron–hole energy densities can be expressed through phonon and electron temperatures, respectively. While the lattice energy density depends only on the lattice temperature, the electron–hole energy density depends also on the carrier density and band gap energy, apart from the electron temperature^29^.

The attenuation of the laser pulse inside the sample is described as^29^:

4 $$\frac{d{I}_{\text{abs}}}{dz}=-\left({\alpha }_{\text{SPA}}+{\alpha }_{\text{FCA}}\right){I}_{\text{abs}}-{\beta }_{\text{TPA}}{I}_{\text{abs}}^{2}$$

with the initial condition in a burst mode

5 $${I}_{\text{abs}}\left(z=0,t\right)=\sqrt{\frac{4\text{ln}\left(2\right)}{\pi }}\left(1-R\right)\frac{{F}_{0}}{\tau }\frac{1}{n}\sum _{j=0}^{n-1}exp\left\{-4\text{ln}\left(2\right){\left[\frac{t-\left(j\Delta t+{t}_{0}\right)}{\tau }\right]}^{2}\right\},$$

where $R$ denotes the reflectivity, ${F}_{0}$ is the peak laser fluence defined as a function of the burst pulse energy ${E}_{\tt{burst}}$ and the laser spot radius $w$ on the sample surface

6 $$ {F}_{0}=\frac{2{E}_{\tt{burst}}}{\pi {w}^{2}}$$

with $\tau$ being the duration of the laser pulse that is centered around ${t}_{0}=2.5\tau$ and $\Delta t$ the time delay between sub-pulses. We take into account only the depth dependence of the absorbed fluence focusing our attention on the center of the laser spot.

Drastic changes of a carrier density during and after irradiation crucially influence the material optical properties such as ${\alpha }_{\text{FCA}}$ and reflectivity. Analogously to Ref.^29^, we apply a Drude model for the complex dielectric function^29,39,40^:

7 $$\varepsilon \left( {\omega _{L} } \right) = \varepsilon _{r} \left( {\omega _{L} } \right) - \frac{{n_{e} e^{2} }}{{\varepsilon _{0} \omega _{L} }}\left[ {\frac{1}{{m_{{e,{\text{cond}}}}^{*} \left( {\omega _{L} + iv_{e} } \right)}} + \frac{1}{{m_{{h,{\text{cond}}}}^{*} \left( {\omega _{L} + iv_{h} } \right)}}} \right]$$

with ${\varepsilon }_{r}$ being the intrinsic dielectric constant, *e* is the electron charge, ${\varepsilon }_{0}$ is the permittivity in vacuum, ${m}_{e,\text{cond}}^{*}$ and ${m}_{h,\text{cond}}^{*}$ are effective masses, ${v}_{e}$ and ${v}_{h}$ are collision frequencies. All details about the construction of the dielectric function are given in Ref.^29^. By knowing the dielectric function, we can calculate the complex refractive index $\stackrel{\sim }{n}={n}_{\text{ind}}+i{k}_{\text{ind}}$, and, therefore, the reflectivity and the FCA coefficient under normal incident irradiation as

8 $$R=\frac{{\left({n}_{\text{ind}}-1\right)}^{2}+{k}_{\text{ind}}^{2}}{{\left({n}_{\text{ind}}+1\right)}^{2}+{k}_{\text{ind}}^{2}},$$

9 $${\alpha }_{\text{FCA}}=\frac{2{\omega }_{L}{k}_{\text{ind}}}{c},$$

where $c$ denotes the speed of light in vacuum.

## Results

### Numerical solution of the nTTM

The nTTM, Eqs. (1)–(3), have been solved numerically with initial conditions of room-temperature (300 K) for electrons as well as phonons, a free-carrier density of 10^12^ cm^−3^ and vanishing boundary conditions $J_{z} \left( {z,0} \right) = 0, \;W_{z} \left( {z,0} \right) = 0$ and ${\kappa }_{ph}\partial {T}_{ph}/\partial z=0$ at the surface $z=0\,\upmu \text{m}$ and at $z=10\,\upmu \text{m}$, assumed as the thickness of the material. The detailed description of the simulation scheme is given in Ref.^29^. We have applied a 200 fs laser pulse with the burst energy ${E}_{\tt{burst}}=0.84\,\upmu \text{J}$, the laser spot radius $w=18\,\upmu\text{m}$ at 1030 nm wavelength. It should be mentioned that the nTTM model is valid in a low-fluence regime, when ${T}_{ph}$ does not overcome the melting threshold.

**Figure 1:**
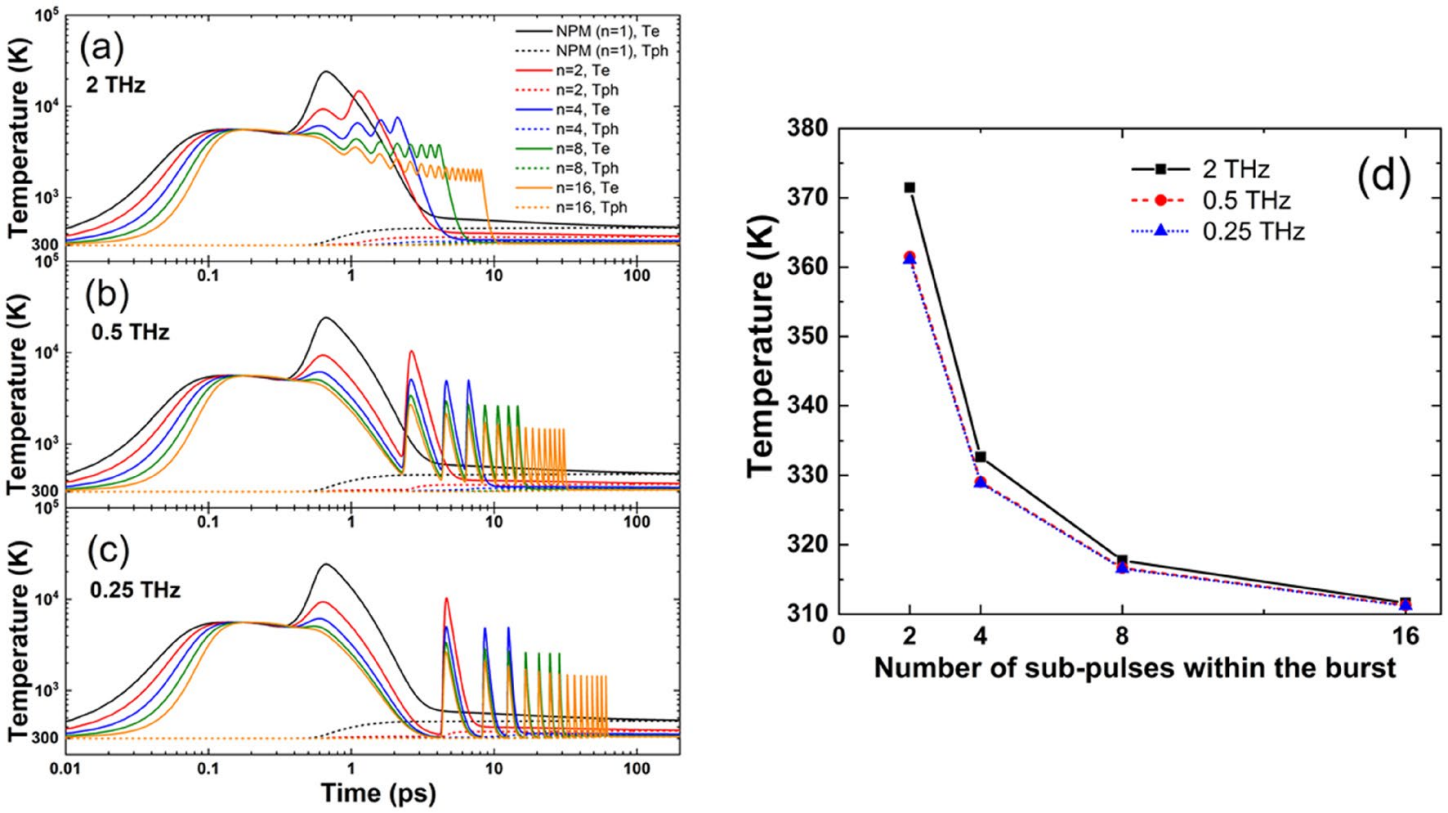
Electron and phonon temperatures at the surface of laser-irradiated silicon obtained with the nTTM with intra-burst frequencies (**a**) 2 THz, (**b**) 0.5 THz, (**c**) 0.25 THz. (**d**) Temperature at the surface *z* = 0 nm at 200 ps (when thermalization between the electron and phonon system was reached) as a function of the number of sub-pulses within the burst for different intra-burst frequencies.

Figure 1a–c represent the temporal evolution of the electron and phonon temperatures at the surface of the laser-irradiated silicon with different numbers of sub-pulses and intra-burst frequency. The electron temperatures start to increase early during the action of the laser pulse until they reach the “plateau” region at about 0.1 ps. This first increase is related to the linear absorption processes^29^. The second rise is partially due to the activation of nonlinear absorption, namely the two-photon absorption. Additionally, the importance of free carrier absorption is increasing, due to the increasing free carrier density. The electron temperatures approach their maxima slightly after the maxima of the laser pulse and then they decrease due to lattice heating. If the number of sub-pulses *n* > 1, then additional rises of electron temperatures are observed.

It can be seen from Fig. 1a that with increasing number of sub-pulses:

- the initial increase of the electron temperature is slower (the electron sub-system is heated slower);
- the maximum electron temperature *T*_*e*_ reached is decreasing;
- the final value of the phonon temperature *T*_*﻿p﻿h*_ is decreasing.

The trends of *T*_*e*_ and *T*_*p﻿﻿h*_ at intra-burst frequencies of 0.5 THz (Fig. 1b) and 0.25 THz (Fig. 1c) are similar to 2 THz. In Fig. 1d, we explicitly show the dependence of the lattice temperature on the number of sub-pulses at 200 ps after the first pulse, which is sufficient to ensure thermalization between electrons and phonons for each burst configuration. The most obvious trend is that the lattice temperature is decreasing with increasing number of sub-pulses. We observe this behavior for each investigated intra-burst frequency. Further, it can be noticed that the lattice temperature is decreasing with decreasing the intra-burst frequency. Note that this is not an effect of the temporal distance to the last pulse in the sequence. Both observations support the experimental findings, which are shown in Fig. 6.

**Figure 2:**
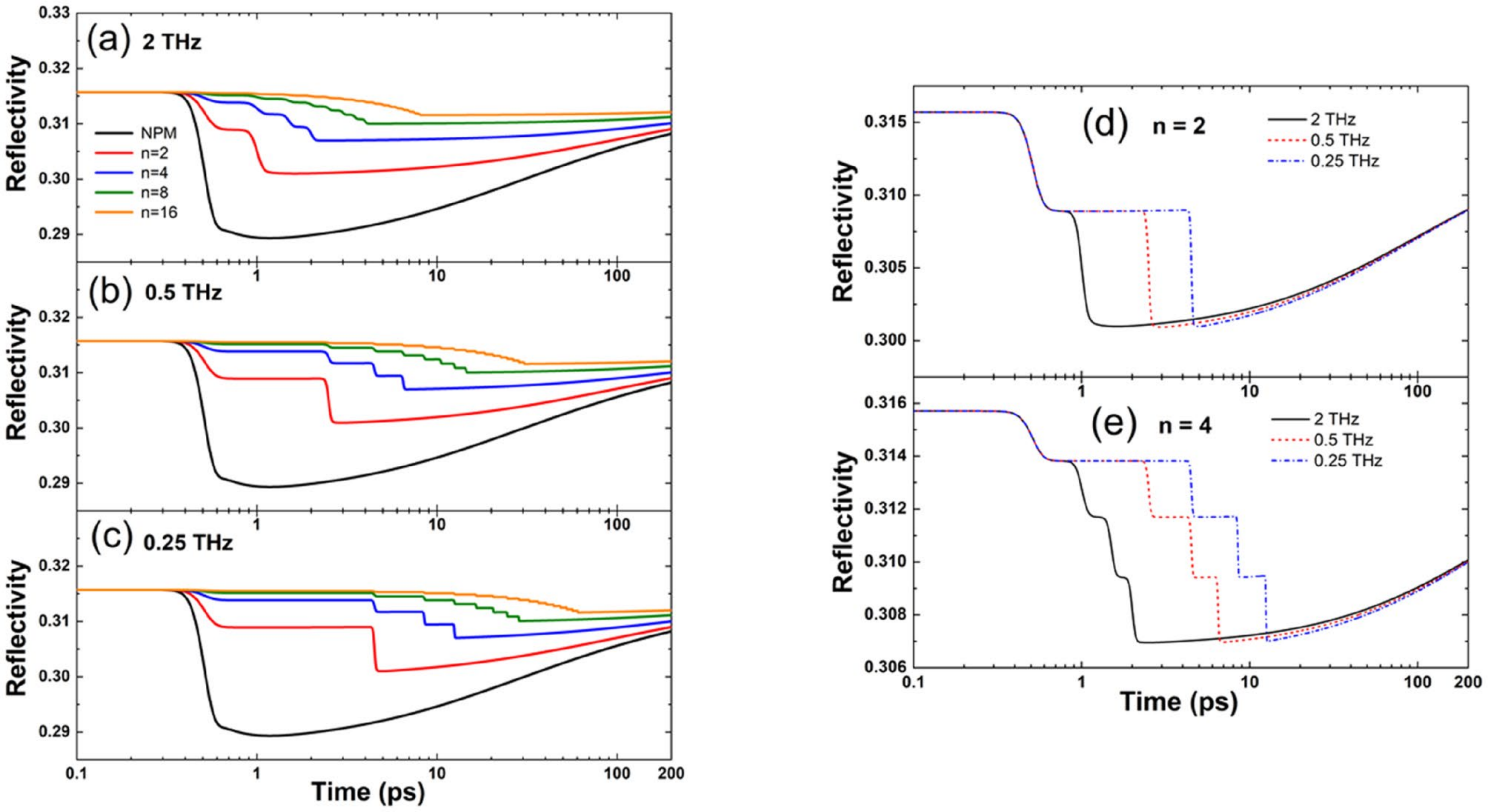
Reflectivity at the surface *z* = 0 nm as a function of time for intra-burst frequencies (**a**) 2THz, (**b**) 0.5 THz, (**c**) 0.25 THz. Comparison of reflectivity at the surface *z* = 0 nm as a function of time for different intra-burst repetition rates with (**d**) *n* = 2 and (**e**) *n* = 4 sub-pulses.

To gain a deeper insight into the laser-matter interaction with irradiation in BM, we investigated the evolution of the reflectivity. In Fig. 2a–c, we present the dependence of the reflectivity on the number of sub-pulses for different intra-burst frequencies. The reflectivity for each configuration falls down during acting of the laser pulses because of the increase of the free carrier density, which influences the dielectric function (see Eqs. (7) and (8)). The number of the drops and time delays depend on the corresponding burst configuration. The drop experienced by the reflectivity is smaller, when increasing the number of pulses in the bursts, because we generate less free carriers. After the burst laser pulse is gone, the reflectivity increases again due to the decay of free carriers. These statements hold for each considered number of sub-pulses and intra-burst frequency.

In Fig. 2d,e, we compare the temporal evolution of the reflectivity for different intra-burst frequencies with a fixed number of sub-pulses, namely *n* = 2 and *n* = 4 sub-pulses. The number of drops for each reflectivity and time delays between them are in accordance with the burst-pulse configuration. We can observe that the depths of the reflectivity drops are approximately the same for the three examined frequencies for a fixed number of sub-pulses, whereas the minimum of reflectivity shifts towards later times, where the maximum of the electron temperature is located. Moreover, it should be mentioned that with decreasing intra-burst frequency, the value of the reflectivity stays higher for longer time.

**Figure 3:**
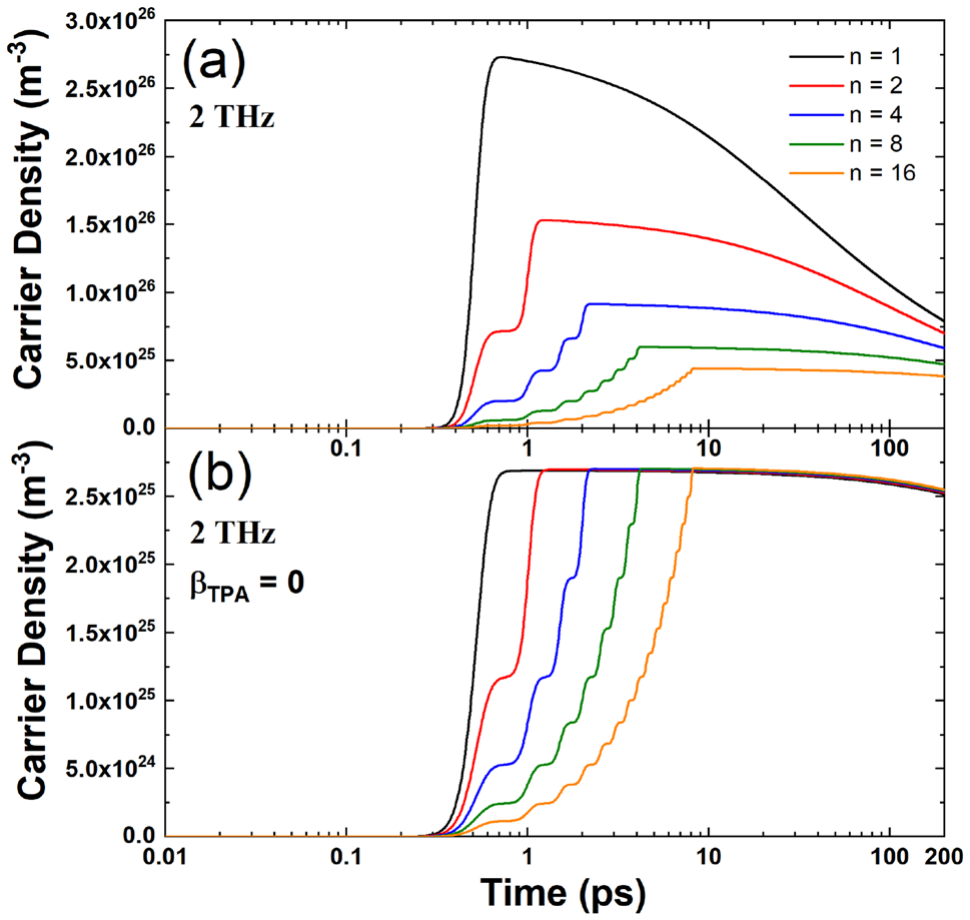
Carrier density at the surface of laser-irradiated silicon obtained in the framework of the nTTM with (**a**) $${\beta }_{\text{TPA}}\ne 0
$$ and (**b**) $${\beta }_{\text{TPA}}=0$$.

It is important to note that Eq. (4) for the attenuation of the laser pulse is strongly nonlinear. To visualize the influence of this nonlinearity, we have set ${\beta }_{\text{TPA}}=0$. The results are presented in Fig. 3 for an intra-burst frequency of 2 THz, where we show the free carrier densities as a function of time at the surface of laser-irradiated silicon. The dynamics of the free carrier evolution is fully governed by the burst-pulse configuration. The rises of the carrier densities are mainly due to one and two photon absorption processes, while they decay because of Auger recombination and carrier transport towards the depth of the sample.

It is seen from Fig. 3a that with increasing number of sub-pulses and ${\beta }_{\text{TPA}}\ne 0$:

- the maximum ${n}_{e}$ decreases significantly;
- the maximum ${n}_{e}$ shifts towards longer times;
- Even at *t* = 200 ps, ${n}_{e}$ with *n* = 16 is lower than ${n}_{e}$ with *n* = 1.

The results from Fig. 3b for ${\beta }_{\text{TPA}}=0$ indicate clearly that there are almost no differences in the maximum for ${n}_{e}$ as *n* increases. Therefore, the reason for different burst features leading to very different carrier densities, lies in the significant influence of the two photon absorption process. Thus, the response of the material to the laser radiation is changing drastically depending on ${\beta }_{\text{TPA}}$.

### Experimental evaluation of BM processing

**Figure 4:**
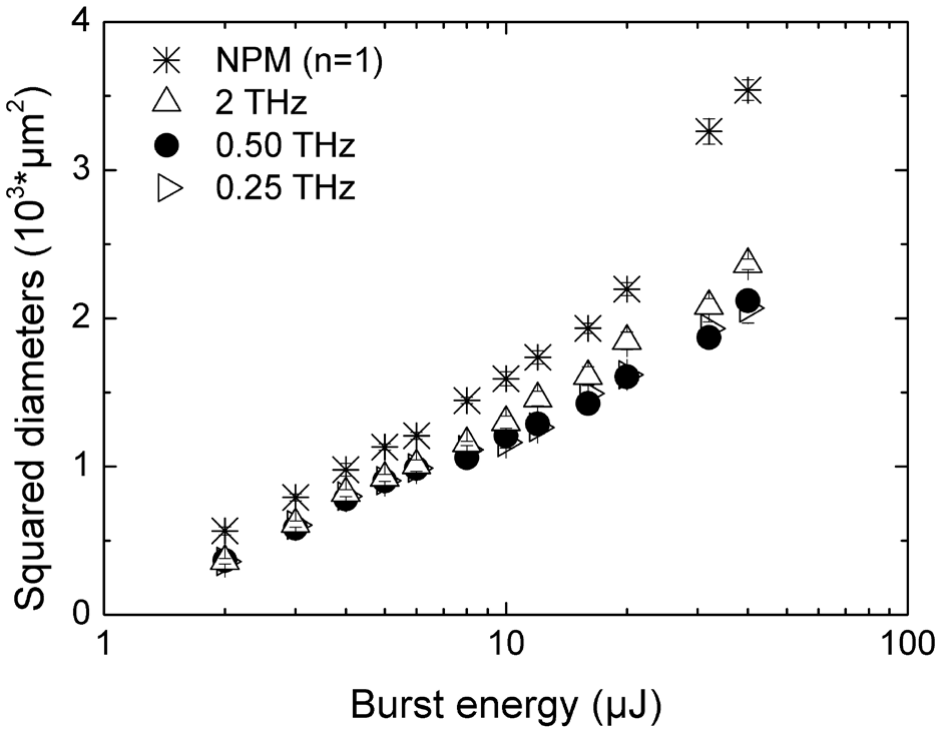
Squared diameters of the ablated craters performed on Si [100] samples versus burst energy, for bursts with 2 sub-pulses at three different intra-burst repetition rates. Results obtained in case of NPM are reported for comparison and indicated by the asterisk.

For the experimental investigation of the burst processing we first plot the square of the mean value of the crater diameters measured for each irradiation condition as a function of the burst energy. This way of representing the experimental results is often used to derive the material laser damage threshold through the linear interpolation of the data and hence study how such physical quantity changes depending on the irradiation conditions^7,41^. Also, this type of plot is useful to distinguish between different ablation regimes since a change of the slope of the data is expected when a transition towards thermal ablation occurs at higher pulse energies^21,42,43^. In Fig. 4, results obtained with bursts with *n* = 2 sub-pulses and different intra-burst frequencies are shown and compared to the NPM case.

It can be noticed that, as expected, the crater diameters increase with the burst energy, regardless of the number of sub-pulses within the bursts. Compared to NPM, the craters produced by bursts of equivalent energy are always smaller. However, what is more interesting to note is that while in the case of NPM a change of the slope is clearly visible at a pulse energy around 20 µJ, this change cannot be seen for the data relating to BM. Although Fig. 4 refers just to the case of *n* = 2, however the same behavior, i.e. no change of the slope, was found for the other investigated burst features. Therefore, it is plausible that splitting the pristine pulse energy into sub-pulses, i.e. BM processing, might be beneficial to prevent thermal side effects and reduce heating of the substrate at higher pulse/burst energies, thus inhibiting the ignition of strong ablation regime^43^, as also suggested by the simulation results presented in Fig. 1. However, it is worth noticing that in recent works^44,45^ Kudryashov et al. have suggested that the origin of such change of in the slope of the craters size versus the applied energy could also be due to a transition towards 3D ablation process. In this case, the lower slope was attributed to non-linear absorption, i.e. wavelength-dependent multiphoton absorption, while the higher slope was ascribed to the linear absorption of the near-critical electron–hole plasma which instead is independent of the wavelength.

**Figure 5:**
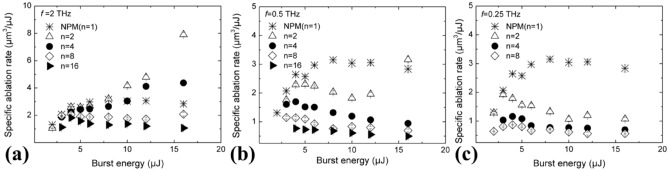
Specific ablation rate as a function of the burst energy, in case of bursts of 2, 4, 8, and 16 pulses at (**a**) 2 THz, (**b**) 0.5 THz and (**c**) 0.25 THz. NPM is shown for comparison.

In order to investigate whether BM processing with such high intra-burst frequencies enables higher ablation rates than NPM, the specific ablation rate, defined as the volume of the craters divided by the burst energy and the number of bursts, has been plotted in Fig. 5 as a function of the burst energy, for the three intra-burst repetition rates.

Here, it is visible that when a high number of sub-pulses and lower intra-burst frequencies are used, after reaching a peak at burst energy lower than 5 μJ, the specific ablation rate decreased, in good agreement with Neuenschwander et al.^9^ However, for lower number of sub-pulses, after reaching a plateau, a new increase of the specific ablation rate can be seen (see for example *n* = 2 and *n* = 4 at 2 THz and *n* = 2 at 0.5 THz), until it also exceeds the value found in NPM.

In fact, it can be noticed that with intra-burst frequencies equal to or lower than 0.5 THz, the specific ablation rate found in NPM is generally higher than in BM, at equal total energy. However, at the highest intra-burst frequency of 2 THz, there are suitable combinations of burst parameters, i.e. *n* = 2 and *n* = 4 sub-pulses and a total burst energy above 10 µJ, that produce a higher specific ablation rate than NPM. For *n* = 2 and a burst energy of 16 µJ, the ablation rate is almost 3 times higher than in NPM.

**Figure 6:**
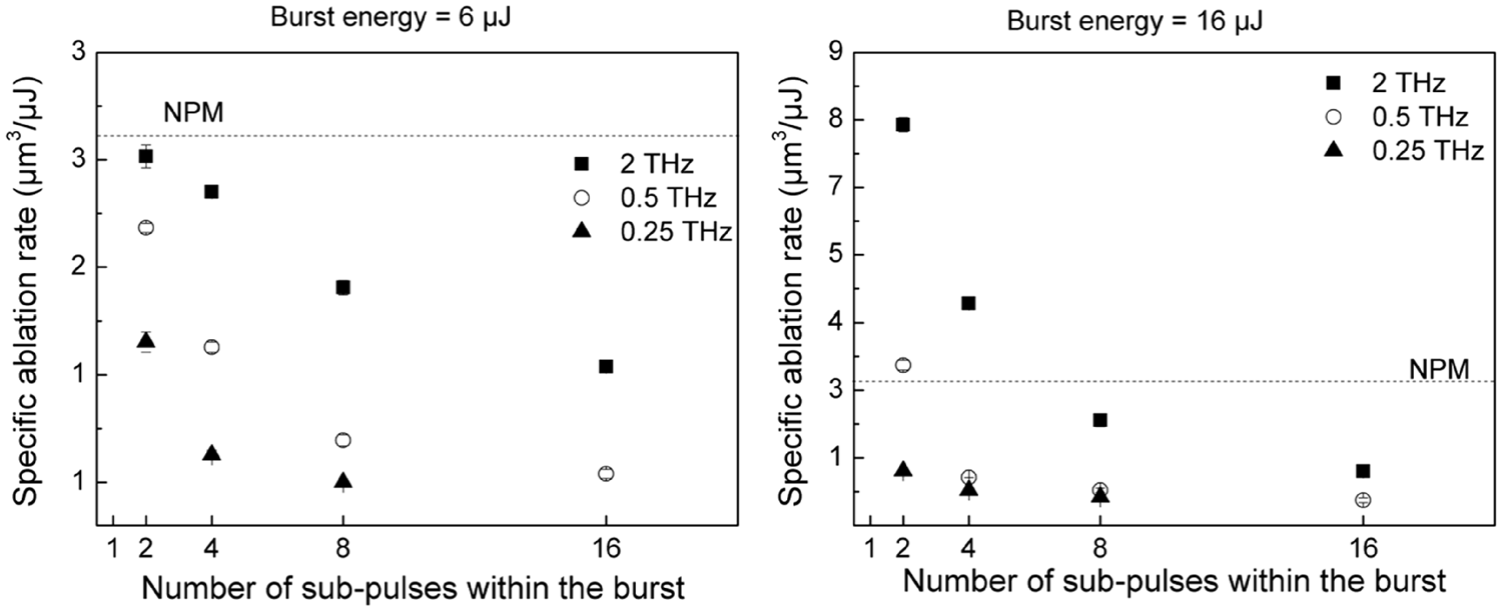
Specific ablation rate versus the number of pulses within the burst, at a burst energy of 6 µJ (left) and 16 µJ (right). The specific ablation rate obtained in NPM is shown by the dashed line.

However, the specific ablation rate decreases as the number of pulses within the burst increases, as it is better visible in Fig. 6 for two different values of the burst energy. When changing the number of pulses from 2 to 16 at 2 THz, the reduction of the specific ablation rate is around 50% at a burst energy of 6 µJ and higher than 85% at 16 µJ. It can be also noticed that a similar decreasing trend was found for all the investigated intra-burst repetition rates.

Moreover, for a given number of pulses, the specific ablation rate decreases, when the intra-burst frequency decreases. Indeed, for *n* = 2 and a burst energy of 6 µJ, a reduction of the specific removal rate around 40% has been registered, when the frequency was decreased from 2 to 0.25 THz. At 16 µJ, the reduction observed going from 2 to 0.25 THz was even more than 80%.

### Discussion

The experimental results of laser ablation of silicon with bursts of femtosecond pulses with frequencies in the THz range find sound physical explanations from the numerical solutions of the nTTM. In particular, it is shown in Fig. 1 that the final temperature reached after thermalization of the electron and the phonon subsystems is lower as *n* increases, which can explain the experimental results reported in Fig. 4. Here, no change of the slope of the plotted data is seen in case of BM processing thus indicating that the thermal ablation regime established in NPM above a certain laser fluence does not occur in BM (at least for the parameter regime investigated). Therefore, a lower thermal load to the material and, thus, lower phonon temperature is expected. This is also confirmed by the simulated temporal evolution of the phonon temperature as shown in Figure S1 of the Supplementary Information, where it is clearly visible that the phonon temperature, at the time when thermalization is reached, is lower for higher number of pulses (see also Fig. 1d).

Except for a narrow process window at 2 THz of intra-burst frequency and *n* = 2 or *n* = 4 pulses, the specific ablation rate achieved in BM processing was generally lower than in NPM with equivalent energy (see Fig. 5a). This can be ascribed to the dynamic changes of the optical properties of silicon during irradiation with bursts at such extremely high repetition rates. The reflectivity drop of the material is, indeed, lower in BM than in NPM processing, as shown in Fig. 2. This indicates a weaker absorption of the incident laser energy explaining the lower ablation rate generally found in BM compared to NPM.

Comparing the results with different burst features in Fig. 6, it can be observed that the specific ablation rate decreases with the number of sub-pulses in the burst. Moreover, a significant reduction was also found at a given number of pulses, when the intra-burst frequency was decreased, especially for a small number of sub-pulses. Both trends find explanations from the simulations of phonon temperature and reflectivity. In fact, the final temperature reached by the lattice is lower as *n* increases (see Fig. 1d) as well as the drop experienced by the reflectivity is smaller, when increasing *n*, as shown in Fig. 2. Therefore, for a higher number of sub-pulses more laser energy is reflected on the surface and less laser energy is absorbed and transferred to the material. Regarding the influence of the intra-burst frequency, from Figures S1 and Fig. 1d it can be noticed that the maximum phonon temperature is higher for higher intra-burst repetition rates for each investigated number of sub-pulses. In addition, it should be mentioned that with increasing time delay the value of the reflectivity stays higher for longer time. In general, higher reflectivity does not necessarily lead to weaker absorption. However, both trends indicate a weaker transfer of the laser energy to the irradiated material, when a longer time delay between sub-pulses (i.e. lower intra-burst frequency) is used, as shown in Figure S2, which is consistent with the experimental trend of the burst specific ablation rate depending on the intra-burst frequency. This behavior can be ascribed to nonlinear absorption processes. In Fig. 3, it was shown that different bursts configurations lead to significantly distinct carrier densities, when the two photon absorption process is considered. Whereas almost no differences can be noticed when it is neglected. Therefore, a key role in changing the optical transient properties of the material and, therefore, its response to irradiation, is played by nonlinear processes. In fact, the final temperature reached by the lattice is lower as n increases (see Fig. 1d) as well as the drop experienced by the reflectivity is smaller, when increasing n, as shown in Fig. 2. Therefore, for a higher number of sub-pulses more laser energy is reflected on the surface and less laser energy is absorbed and transferred to the material. Regarding the influence of the intra-burst frequency, from Figures S1 and Fig. 1d it can be noticed that the maximum phonon temperature is higher for higher intra-burst repetition rates for each investigated number of sub-pulses. In addition, it should be mentioned that with increasing time delay the value of the reflectivity stays higher for longer time. Both trends indicate a weaker transfer of the laser energy to the irradiated material, when a longer time delay between sub-pulses (i.e. lower intra-burst frequency) is used, as shown in Figure S2, which is consistent with the experimental trend of the burst specific ablation rate depending on the intra-burst frequency.

## Conclusion

A theoretical and experimental study on the laser ablation of crystalline silicon with bursts of femtosecond pulses with THz intra-burst repetition rates has been carried out. The ultrashort pulsed laser–matter interaction phenomena occurring at such extreme and almost unexplored burst frequencies, i.e. where the temporal separation between the pulses is of the order of the electron–phonon coupling time, have been investigated. Craters were ablated on silicon samples using fixed numbers of bursts of 200-fs pulses, where the total burst energy, the number of sub-pulses in the burst and the intra-burst frequency were systematically varied. Based on the measurements of the crater depths and diameters it was possible to estimate the specific ablation rate for each irradiation condition. It was found that, compared to normal pulse mode (NPM), burst mode (BM) processing allows reducing the thermal load on the substrate. This experimental result has found confirmation from the simulations of the temporal evolution of the lattice temperature calculated on the basis of the numerical solution of the density-dependent two temperature model, where it was shown that the temperature reached by the substrate decreases with the pulse splitting in the burst. It was further observed that the specific ablation rate reduced as the number of sub-pulses increased. This trend was explained in terms of the change of reflectivity of the material depending on the irradiation conditions. According to the simulations, samples irradiated with a burst with higher number of sub-pulses experienced a weaker reflectivity drop, which means that less energy was transferred from the laser to the material. Although less pronounced, the same happened when the time delay between sub-pulses in the burst was longer, i.e. the intra-burst frequency was lower. Correspondingly, the highest values of the experimentally measured specific ablation rate were observed, when bursts with 2 THz frequency and low number of sub-pulses (*n* = 2) were used.

Generally, NPM performed better than BM in terms of specific ablation rate and energy transfer efficiency. However, a narrow experimental process parameter window (i.e. *n* = 2 or 4 and 2-THz intra-burst frequency, total burst energy above 10 µJ) has been found, where BM processing ablates more efficiently compared to NPM. This result does not find explanation from the numerical simulations. In order to explain this theoretically, further investigations including the description of phase transitions are needed. In addition, at high laser fluences the reflectivity dynamics can experience more complex behavior^46,47^, for example, initial rise followed by a decay in contrast with the initial decrease accompanied by the rising observed in our work. Moreover, simulation results definitely suggest that non-linear processes play a fundamental role during laser irradiation of silicon with bursts of ultrashort pulses in the THz frequency range. In particular, the two photon absorption process has been found to have significant influence on the carrier density temporal evolution. Thus, the response of the material to the laser radiation is changing drastically depending on its two-photon absorption coefficient value. This would imply a significant wavelength dependence, which is worth being investigated in the future.

## Supplementary Information

Supplementary Information 1.

## Data Availability

The datasets generated during and/or analyzed during the current study are available from the corresponding authors on reasonable request.
