# Supplementary material for: Laser ablation of silicon with THz bursts of femtosecond pulses
Source: Sci Rep. 2021 Jun 25;11:13321. doi: 10.1038/s41598-021-92645-7 (PMC8233330; doi:10.1038/s41598-021-92645-7)
Supplement: Supplementary file 1 — Supplementary Information 1. [file 41598_2021_92645_MOESM1_ESM.pdf]

SUPPLEMENTARY INFORMATION

Laser ablation of silicon with THz bursts of femtosecond pulses

Caterina Gaudiuso[1,2,*,#], Pavel N. Terekhin[3,*,#], Annalisa Volpe[1,2], Stefan Nolte[4,5], Baerbel Rethfeld[3], Antonio Ancona[1,2]

[1] Department of Physics, University of Bari "Aldo Moro," Bari 70126, Italy

[2] CNR-IFN UOS BARI, Via Amendola 173, Bari, Italy

[3] Department of Physics and Research Center OPTIMAS, Technische Universität Kaiserslautern, Erwin-Schrödinger-Strasse 46, 67663 Kaiserslautern, Germany

[4] Institute of Applied Physics, Abbe Center of Photonics, Friedrich-Schiller-Universität Jena, Albert-Einstein-Strasse 15, 07745 Jena, Germany

[5] Fraunhofer Institute for Applied Optics and Precision Engineering IOF, Center of Excellence in Photonics, Albert-Einstein-Strasse 7, 07745 Jena, Germany

[*]Correspondence and requests should be addressed to C.G. (email: caterina.gaudiuso@uniba.it) and P.N.T. (email: terekhin@physik.uni-kl.de)

[#]Contributed equally to this work

Table of Contents

1. Numerical solutions of nTTM
   (a) Influence of the intra-burst frequency on the phonon temperature
   (b) Energy coupling between THz bursts of fs pulses and silicon.

1. Numerical solutions of nTTM

(a) Influence of the intra-burst frequency on the phonon temperature

Burst Mode (BM) processing proves to be beneficial for reducing the thermal issues related to the heat generation which follows the laser irradiation. The heating of the material is significantly reduced because the pulse energy is split among several sub-pulses with THz intra-burst frequency.

In Fig. S1, the evolution of phonon temperatures is shown for a 2- and 16-sub-pulses burst. We compare in this figure different intra-burst frequencies, ranging from 0.25 THz to 2 THz. The number of rises of the phonon temperature is in accordance with the number of sub-pulses. The final phonon temperatures are almost the same (a bit lower for 0.25 THz, then for 0.5 THz) for the two lower frequencies, which corresponds to sub-pulse delays of 2 ps and 4 ps, respectively. However, at higher intra-burst frequency a higher phonon temperature is reached. These trends hold true for *n*=16 (Fig. S1b) and for other number of sub-pulses (not shown). Therefore, we can conclude that for each investigated number of sub-pulses, the resulting phonon temperature is lower for longer time delays, i.e. lower burst frequencies.

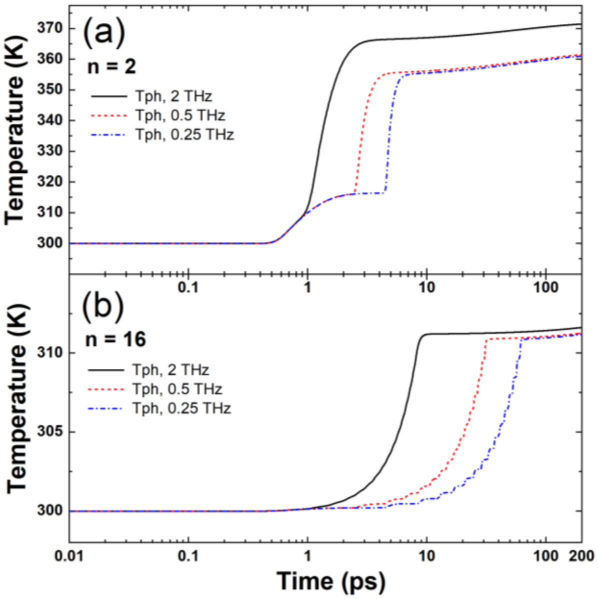

Figure S1. The phonon temperatures at the surface z = 0 nm as a function of time with (a) *n*=2 and (b) *n*=16 sub-pulses at the three investigated intra-burst frequencies.

(b) Energy coupling between THz bursts of fs pulses and silicon

It BM, it is clear that a special role in the laser-matter interaction is played by the non-linear absorption processes, namely the two photon absorption. This results in a weaker reflectivity drop in case bursts are used which caused absorption of the laser radiation to decrease. This is clearly shown in Figure S2 where the total energy densities of laser-irradiated silicon are shown. Here, we can see that for *n*=2 the shapes of the energy density curves (number of rises and time delay between them) are defined by the given burst irradiation configuration. The final absorbed energy is the highest for the 2 THz intra-burst frequency, while

the results are almost the same for 0.5 THz and 0.25 THz after the end of the laser pulse sequence. However, the amount of absorbed energy is a little bit higher for the 0.5 THz frequency than for the 0.25 THz frequency. The same trends are seen in Fig. S2 b for *n*=16.

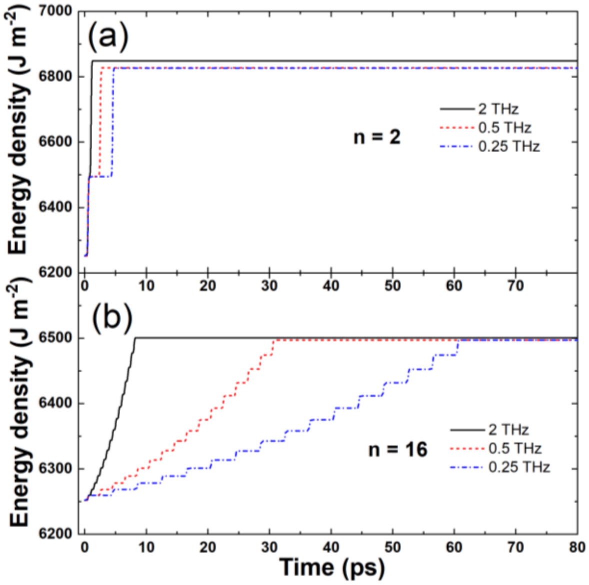

Figure S2. Total energy density of laser-irradiated silicon obtained with the nTTM with (a) *n*=2 and (b) *n*=16 sub-pulses and different intra-burst repetition rates.

It can be concluded from Fig. S2 that less energy is transferred from the laser to the material when a higher number of sub-pulses is used. The same holds true for a longer time delay between the sub-pulses (lower burst frequency), but this is much less pronounced.
